# Depression, Anxiety, and Stress Among Final-year Medical Students

**DOI:** 10.7759/cureus.4257

**Published:** 2019-03-16

**Authors:** Besham Kumar, Mir Ali Asghar Shah, Raj Kumari, Ajay Kumar, Jai Kumar, Amber Tahir

**Affiliations:** 1 Internal Medicine, Jinnah Postgraduate Medical Center, Karachi, PAK; 2 Internal Medicine, People's University of Medical and Health Sciences for Women, Karachi, PAK; 3 Internal Medicine, Jinnah Sindh Medical University, Karachi, PAK; 4 Internal Medicine, Liaquat University of Medical and Health Sciences, Karachi, PAK; 5 Internal Medicine, Dow University of Health Sciences, Karachi, PAK

**Keywords:** depression, stress in medical students, medical student research, anxiety, stress, dass-21

## Abstract

Introduction

The overall environment of the medical school is often considered very stressful. It projects negative effects not only on the academic performances of medical students but also deteriorate their physical health and psychosocial wellbeing. The aim of this study was to determine the frequency of depression, stress, and anxiety among final year medical students.

Methods

This observational study was conducted in public and private medical colleges in February 2019. The instrument utilized in this study was Depression, Anxiety, and Stress Scale (DASS-21). Factors predisposing to depression, stress, and anxiety were also recorded. Data were entered and analyzed using SPSS v. 21.

Results

The mean scores of depression, anxiety, and stress were 18.00 ± 11.5, 19.15 ± 11.2, and 20.92 ± 11.2, respectively. The mean score of anxiety and stress was higher in private college students, while that of depression was higher in public college students. Overall, 57.6% of the students suffered from moderate to extremely severe depression, 74% of the students suffered from moderate to extremely severe anxiety, and 57.7% students had moderate to extremely severe stress. The common reasons to high stress and anxiety included the pressure of passing exams, the pressure of living up to family’s expectations, fear of stepping into the real world of medicine, and dissatisfaction with the administration.

Conclusion

The incidence of psychological illnesses including anxiety, stress, and depression is high among the medical students of Pakistan. Reasons predisposing the students to these illnesses must be efficiently tackled.

## Introduction

The overall environment of the medical school is often considered very stressful that projects negative effects not only on the academic performances of medical students but also deteriorate their physical health and psychosocial wellbeing [1]. There is growing concern worldwide regarding the psychological health of students particularly medical students. They are said to be more vulnerable to depression and anxiety.

Various factors such as academic burden, highly competent peers, hindrances in reaching their desired specialty, and transition phase from being a school student to almost being a physician play important role in affecting their psychological health [2-3]. Other factors include undue expectations from self, family members, and also teachers while being trained to take responsibility for the well-being and life of patients also contributes to stress among medical students [4]. The above-mentioned factors combined with the fact that because of a high amount of time needed to complete academic studies, students are not able to participate in extracurricular activities such as pursuing their hobbies [5]. This increasing stress level among the medical students, in the long term, can have many deleterious effects on them including poor academic performance and poor quality of life [6]. All these factors may be the reason that medical students, when compared to the general population of the same age group, were found to have higher levels of depression and anxiety [7-8].

There is also reported difference between depression and anxiety levels of students attending public and private medical school. Medical students all across the globe have shown increase level of stress and depression including countries like Canada and Malaysia [8,9]. However, little attention has been paid towards anxiety and stress among medical students in Pakistan. The aim of this study is to evaluate the degree of anxiety, stress, and depression among final-year medical students.

## Materials and methods

This observational study was conducted in two medical colleges of Karachi - Jinnah Sindh Medical University (public institute) and Ziauddin Medical College (private institute) - in February 2019. All final-year students from both colleges were invited to participate (N = 450). There were 312 final-year medical students who completed the study (response rate: 69.3%).

An online proforma was circulated, which included Depression Anxiety Stress Scale-21 (DASS-21). DASS-21 is a 21-item self-reported instrument designed to measure the three related negative emotional states including depression, anxiety, and stress. It has 21-items, with seven items for each subscale. Students scored each item from 0-3, where zero meant "did not apply to me at all" and three meant "applied to me very much". All scores of each subscale were added and multiplied by two. "Normal" score for depression was 0-9, for anxiety 0-7, and for stress 0-14. "Mild" score for depression was taken 10-13, for anxiety 8-9, and for stress 15-18. "Moderate" score for depression was 14-20, for anxiety 10-14, and for stress 19-25. "Moderate" score for depression was 14-20, for anxiety 10-14, and for stress 19-25. "Severe" score for depression was 21-27, for anxiety 15-19, and for stress 26-33. "Extremely severe" score for depression was 28+, for anxiety 20+, and for stress 34+ [10]. Validity and reliability of the Iranian version of DASS-21 were determined on a good level by the previous study (Cronbach Alpha 0.77, 0.79, and 0.78 for depression, anxiety, and stress domains, respectively) [11]. The online proforma also included reasons predisposing students to anxiety, stress, and depression. The students could choose as many factors as applicable to them.

Data were analyzed using IBM SPSS Statistics for Windows, Version 21.0. Armonk, NY: IBM Corp. Internal consistency of DASS-21 as a whole and each of its subscale was calculated by Cronbach alpha. The mean and standard deviation (SD) were calculated for the scores of anxiety, stress, and depression. Frequencies and percentages were calculated for the severity of DASS-21 and reasons predisposing students to anxiety, stress, and depression.

## Results

Out of 312 final-year medical students who participated in the study, 264 (84.6%) were women and 48 (15.4%) were men. Their mean age was 22.74 ± 1.52 years. The overall mean score of anxiety was 19.15 ± 11.2; depression 18.00 ± 11.5; and stress 20.92 ± 11.2. There were 57.6% participants who suffered from moderate to extremely severe depression, 74% participants who suffered from moderate to extremely severe anxiety, and 57.7% participants who reported moderate to extremely severe stress.

Cronbach alpha for DASS-21 in our sample was 0.71; for subscale anxiety, it was 0.68; for depression 0.76; and for stress, it was 0.72. The mean anxiety score and the severity of anxiety categorized according to the gender and type of institute are shown in Table 1. In females, not only the mean anxiety score was higher, but females also had more severe anxiety. The students of private medical college reported higher anxiety scores, as seen in Table 1.

**Table 1:** Mean anxiety score and severity of anxiety categorized according to gender and the type of institute Normal, 0-7; mild, 8-9; moderate, 10-14; severe, 15-19; extremely severe, 20+ SD, standard deviation

Demographic characteristic	Mean ± SD	Severity n (%)				
		Normal	Mild	Moderate	Severe	Extremely severe
Gender						
Male	13.00 ± 10.3	24 (50%)	12 (25%)	12 (25%)	--	--
Female	20.27 ± 11.1	36 (13.6%)	24 (9.1%)	--	48 (18.2%)	156 (59.1%)
Type of institute						
Private	21.33 ± 4.1	--	--	--	12 (33.3%)	24 (66.7%)
Public	18.87 ± 11.8	60 (21.7%)	24 (8.7%)	12 (4.3%)	36 (13%)	144 (52.2%)

The mean score of stress and its severity from mild to extremely severe categorized according to the gender and type of institute are shown in Table 2. The mean stress score was higher in females. Three-fourth of the male students were not stressed and only one-fourth were moderately stressed. Although the mean stress score was higher in students of the private medical college, none of them were extremely stressed in comparison to public college students where 17.4% were extremely stressed as seen in Table 2.

**Table 2:** Mean stress score and severity of stress categorized according to gender and the type of institute Normal, 0-14; mild, 15-18; moderate, 19-25; severe, 26-33; extremely severe, 34+ SD, standard deviation

Demographic characteristic	Mean ± SD	Severity n (%)				
		Normal	Mild	Moderate	Severe	Extremely severe
Gender						
Male	11.00 ± 7.6	36 (75%)	--	12 (25%)	--	--
Female	22.73 ± 10.8	72 (27.3%)	24 (9.1%)	72 (27.3%)	48 (18.2%)	48 (18.2%)
Type of institute						
Private	23.33 ± 5.1	--	12 (33.3%)	12 (33.3%)	12 (33.3%)	--
Public	20.61 ± 11.8	108 (39.1%)	12 (4.3%)	72 (26.1%)	36 (13%)	48 (17.4%)

When depression scores were categorized for gender, again females scored higher than males. Overall, 50% of males were not depressed, while 27.3% of females were not depressed. However, 13.6% of females were severely depressed and 31.8% were extremely severely depressed, while none of the male students were severely or extremely severely depressed. The mean score of depression was higher in public college students and 13% of these were severely depressed and 30.4% were extremely severely depressed; while none of the students of the private college were severely or extremely severely depressed as seen in Table 3.

**Table 3:** Mean depression score and severity of depression categorized according to gender and the type of institute Normal, 0-9; mild, 10-13; moderate, 14-20; severe, 21-27; extremely severe, 28+ SD, standard deviation

Demographic characteristic	Mean ± SD	Severity n (%)				
		Normal	Mild	Moderate	Severe	Extremely severe
Gender						
Male	11.00 ± 4.1	24 (50%)	12 (25%)	12 (25%)	--	--
Female	19.27 ±11.9	72 (27.3%)	24 (9.1%)	48 (18.2%)	36 (13.6%)	84 (31.8%)
Type of institute						
Private	11.33 ± 2.5	12 (33.3%)	12 (33.3%)	12 (33.3%)	--	--
Public	18.87 ± 11.9	84 (30.4%)	24 (8.7%)	48 (17.4%)	36 (13%)	84 (30.4%)

The trend of factors predisposing students to these psychological illnesses is shown in Table 4. The pressure of passing exams was a very common reason among both private and public college students. Similarly, both groups had the pressure of living up to the expectations of their families. The pressure of studies was more common in private college students (86.1% vs. 55%), while the fear of stepping into the real world of medicine was more common in public college students (84.7% vs. 52.7%). More students of the public college were dissatisfied with their administration than those of private college (93.4% vs. 61.1%), as shown in Table 4.

**Table 4:** Reasons predisposing medical students of public and private medical colleges to anxiety, stress, and depression

Reasons for anxiety, stress, depression	Private college students (n = 36)	Public college students (n = 276)
Pressure of studies	31 (86.1%)	152 (55.0%)
Pressure of passing exams	34 (94.4%)	259 (93.8%)
Pressure to fulfilling family’s expectation	28 (77.7%)	208 (75.3%)
Fear of stepping into the real world	19 (52.7%)	234 (84.7%)
Dissatisfaction with administration	22 (61.1%)	258 (93.4%)
Dissatisfaction with examination criteria	25 (69.4%)	260 (94.2%)
Cluelessness about future choices of specialty	17 (47.2%)	171 (61.9%)
Missing family / away from home	13 (36.1%)	83 (30.1%)

## Discussion

Depression, anxiety, and stress among medical students are often underrecognized and undertreated. Medical students also seldom seek professional help, mostly because of shame and taboo concerning mental health [12]. Anxiety and depression in medical students in Pakistan were significantly higher as compared to the students in western countries. University of Michigan Medical School, in 2010, reported 14.3% of their students to be depressed [13]. The prevalence of depression among medical students in the United Kingdom was reported to be 24% [14]. In comparison, the study reported 57.6% of medical students in Pakistan to be depressed. Another study conducted in Pakistan showed anxiety and depression to be prevalent among 60% of medical students in a private institute and 43% in a public institute [15-16]. Various factors can be attributed to these ratios of higher incidence in this part of the world. There has been reported the correlation of anxiety and depression with dissatisfaction with examination criteria, overburdening test schedule, and the pressure to pass examinations [17-18]. In Asian families, the pressure from the family to choose medicine as a profession is far greater which is then followed by the need to live up to the family’s expectations regarding their academic performance [18].

The next step after finding the prevalence of anxiety, depression, and stress among medical students is to identify the factors that lead to these mental health issues. This can be done by appointment of student and health counselors that can help to identify stressors such as frustration, pressure, and changes in mood and emotions. Identifying these stressors at the early stage in the medical students will prevent students from succumbing to debilitating psychological issues which, if left unattended, eventually lead to graves outcomes including suicidal attempts [19].

Along with counselors, conducting workshops regarding stress management, awareness programs about stresses and mental health services in campuses will give the students sufficient knowledge and insight about depression, anxiety, and stress and how to deal with it amidst the high pressure of medical school. Academic workload in the field of medicine is universal and almost unavoidable; however, students should be enabled to manage their workload and pressure with the help of counselors. In this regard, an Indian interventional study concluded that students who benefited from a counselor showed lower anxiety and depression scores as compared to the control group who was not exposed to mental health counselors [20].

This study has a few limitations. One of the limitations is that this study did not focus on other causes for depression and anxiety such as personality, time of academic year, and family pressure. The second limitation is that the findings of this study may not be generalized as the results are based upon one private university and one public university. Large scale multi-centric studies are required that measure stress, anxiety, and depression levels among medical students across various time of academic year. Barriers that lead to seeking medical help for mental issues by medical students should also be addressed.

## Conclusions

The incidence of psychological illnesses including anxiety and stress are higher in private college students, while that of depression was higher in public college students. More than half of the students suffer depression and stress, and the incidence of anxiety is even higher. The common reasons to high stress and anxiety included pressure of passing exams, pressure of living up to family’s expectations, fear of stepping into the real world of medicine, and dissatisfaction with the administration. This profession is highly demanding and requires utmost focus and expertise. There is a desperate need to take measures to enhance the mental health of medical students who will be the future lifesavers.
